# Li–Fraumeni-associated pancreatic neuroendocrine tumour and *XAF1* p.Glu134Ter risk modifier variant

**DOI:** 10.3332/ecancer.2022.1487

**Published:** 2022-12-08

**Authors:** Rachel P Riechelmann, Diogo C Soares, Carla Dias, Dirce M Carraro, Giovana T Torrezan

**Affiliations:** 1 Department of Clinical Oncology, A.C. Camargo Cancer Center, Rua Antônio Prudente 211, São Paulo, SP 01509‐010, Brazil; 2 Department of Oncogenetics, A.C. Camargo Cancer Center, Rua Antônio Prudente 211, São Paulo, SP 01509‐010, Brazil; 3 Instituto do Câncer do Estado de São Paulo, São Paulo, SP 01246-000, Brazil; 4 Genomics and Molecular Biology Group, International Research Center/CIPE, A.C. Camargo Cancer Center, Rua Antônio Prudente 211, São Paulo, SP 01509‐010, Brazil; 5 National Institute of Science and Technology in Oncogenomics and Therapeutic Innovation, São Paulo, SP 01509‐010, Brazil

**Keywords:** Li–Fraumeni, neuroendocrine tumour, pancreas neoplasm

## Abstract

Studies have demonstrated that up to 17% of patients with pancreatic neuroendocrine tumours (pNETs) present pathogenic germline variants (PGVs) in several different genes, irrespective of family cancer history. Li–Fraumeni syndrome (LFS) is an autosomal dominant cancer predisposition syndrome related to PGVs in the *TP53* gene. A previous case of a pNET associated with LFS (c.1009C > T, p.R337C) has been reported. Here we report the first case of a patient with pNET and *TP53* p.R337H and *XAF1* p.E134* germline variants, expanding the knowledge of LFS and germline mutations in neuroendocrine tumours.

## Introduction

Hereditary genetic syndromes associated with pancreatic neuroendocrine tumours (pNETs) are rare, occurring in nearly 10% of cases, mostly in multiple endocrine neoplasia type 1 (*MEN1*), von Hippel–Lindau disease (*VHL*), neurofibromatosis type 1 or tuberous sclerosis complex 1 and 2 (*TSC1* or *TSC2*). Yet recent studies have shown that monoallelic pathogenic germline variants (PGVs) have been found in 16%–17% of pNET patients, including PGVs in *MUTYH*, *CHEK2* and *BRCA2, *without known classic clinical criteria for suspecting hereditary syndromes [1–3].

Li–Fraumeni syndrome (LFS) is an autosomal dominant cancer predisposition syndrome related to PGVs in *TP53* gene. Several studies have demonstrated a great variety of tumours that arise in individuals with LFS, such as breast cancer, bone and soft tissue sarcomas, adrenocortical carcinomas and central nervous system tumours. Less commonly, LFS patients may develop other tumours such as colorectal and gastric adenocarcinomas, haematological malignancies, thyroid cancer, melanoma, lung and prostate cancers. The majority of *TP53* PGVs are located in p53 DNA-binding domain with patients presenting a highly penetrant phenotype. There is a founder variant c.1010G>A, p.(Arg337His) located in the p53 oligomerisation domain, highly prevalent in Southern and Southeastern Brazil and which is characterised by a wide range of malignancies but with a lower penetrance when compared to other PGVs (15%–20% versus 50% in p.R337H versus classical mutations carriers, respectively, develop cancer by 30 years of age); p.R337H carriers present a higher risk of developing adrenocortical carcinomas, renal tumours, papillary thyroid cancer and lung adenocarcinomas [4]. Interestingly, family cancer histories of carriers of the Brazilian founder *TP53* p.R337H allele may present from isolated cancer cases to those with multiple tumours resembling classic LFS [4]. Recent studies have suggested that a germline nonsense variant E134*/Glu134Ter/rs146752602 in the tumour-suppressor gene X-linked inhibitor of apoptosis (XIAP)-associated factor 1 (*XAF1*) is a genetic modifier of p53 function and may induce a more aggressive cancer phenotype in LFS carriers of *TP53* p.R337H [5].

Only one previous case of a pNET associated with LFS has been reported. The case was of a 43-year-old female with a G2 pNET and a rare *TP53* PGVs on the residue 337, c.1009C > T, p.R337C, which was also found in her pNET tissue with an allele frequency of 93% [6]. Here we report the first case of a patient with pNET and *TP53* p.R337H and *XAF1* p.E134* germline variants.

## Case report

**Figure 1.**
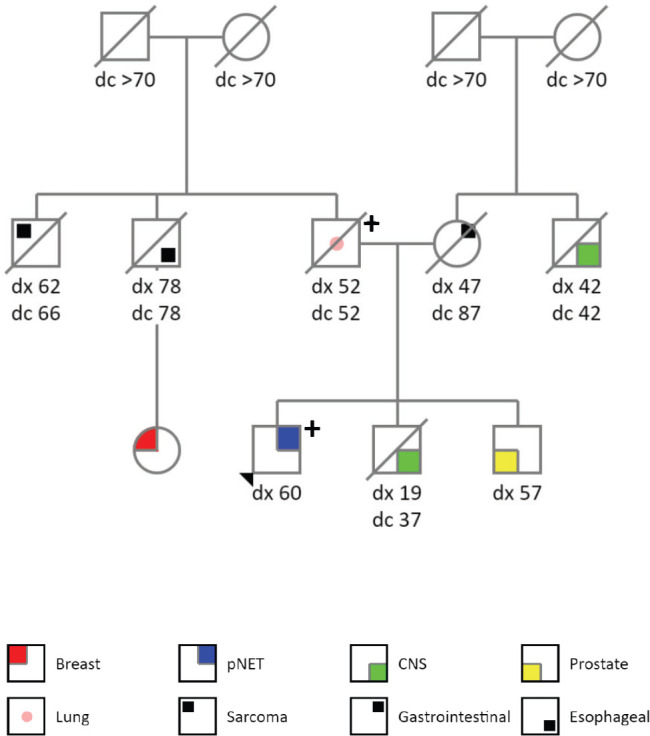
Genogram.

The patient is a 60-years old previously healthy and asymptomatic male who underwent several laboratory and imaging tests to screen out cancer after his father was diagnosed with LFS (PGV in *TP53*, c.1010G>A, p.(Arg337His)). His family cancer history was significant: father with lung adenocarcinoma at the age of 52 years old (died with 52 years), brother with a central nervous system tumour at 19 years (died when 37 years old), brother with prostate cancer at 57 years (alive with 63 years), paternal uncle with oesophageal cancer at 78 years (died with 78 years), paternal cousin with breast cancer at 59 years (alive with 63 years), paternal uncle with soft tissue sarcoma at 62 years (died at 66 years), mother with gastrointestinal cancer at 47 years (died at 87 years), maternal uncle with central nervous system tumour at 42 years (died with 42 years), both grandmothers died with more than 70 years and without cancer history. Figure 1 describes his genogram.

An abdominal ultrasound was performed and identified multiple hypoechoid nodules of varying size, with the largest ones being of 28 × 24 mm and 26 × 18 mm located in segment IV. A magnetic resonance image of the abdomen showed a 3.5 × 2.7 cm nodular lesion in the pancreatic tail and numerous bilobar hepatic lesions measuring up to 5 cm. A liver biopsy was performed in November 2020, of which the pathology showed a well-differentiated grade 3 neuroendocrine tumour infiltrating the liver tissue; tumour cells stained positive by immunohistochemistry for chromogranin A, synaptophysin, CD56 and alpha-antitrypsin, with a ki67 proliferative index of 35%. The ^68^Gallium-DOTATATE positron emission tomography/computed tomography (PETCT) showed positive uptake in all tumour lesions: a pancreatic tail lesion with a standardised uptake value (SUV) of 67.3 and multiple hepatic nodules with SUV maximum of 47. Other imaging and laboratory tests performed to screen out cancer in individuals with LFS did not identified other neoplasms.

**Figure 2.**
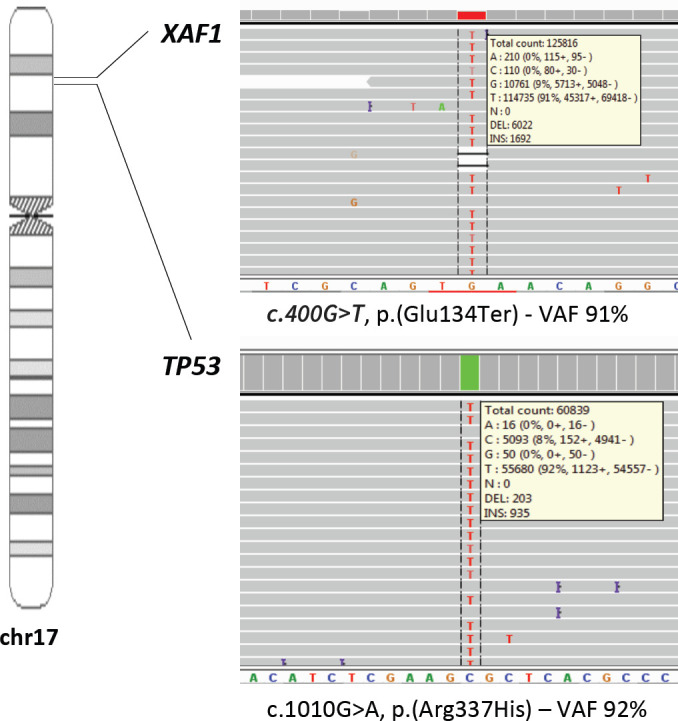
Loss of the wild-type alternate allele (LOH) of TP53 and XAF1 germline variants.

Genetic evaluation was undertaken to confirm the *TP53* p.R337H germline variant and to investigate loss of heterozygosis (LOH) of *TP53* wild allele in the tumour tissue. We found heterozygous PGVs in *TP53* (c.1010G>A, p.[Arg337His]) and in *XAF1* (c.400G>T, p.[Glu134Ter]). Tumour DNA extracted from a paraffin-embedded biopsy of a pNET liver metastasis showed loss of the wild-type alternate allele (LOH) of both germline variants (Figure 2), with allele frequency of 92% for *TP53* and allele frequency of 91% for *XAF1.*

Because the patient was asymptomatic and presented pNET lesions with high SUV in the ^68^Gallium-DOTATE PETCT, we started treatment with octreotide 30 mg long-acting release (LAR) monthly in January 2021. Re-imaging in 4 months demonstrated high-volume progression in liver metastases, weight loss and abdominal pain. Modified FOLFIRINOX was started, and the patient experienced significant clinical benefit after 2 months of treatment: he gained weight, had complete resolution of his abdominal discomfort and radiological imaging reported overall disease stabilisation with minor tumour shrinkage.

Informed consent was provided by the patient to report the case and perform genetic analyses.

## Discussion

We report the first pNET case associated with p.Glu134Ter *XAF1* and p.R337H *TP53* variants*,* documented by LOH in the tumour. This represents a new discovery that expands the clinical spectrum of LFS, particularly for the founder variant LFS highly prevalent in Southern and Southeastern of Brazil.

PGVs in *TP53* gene detected in patients with classical LFS most commonly occur within the DNA-binding domain, leading to the production of a dysfunctional p53 protein [7]. While the oligomerisation domain* TP53* p.R337H variant encountered in our patient is relatively uncommon worldwide, it is predominantly found in Southern and Southeastern of Brazil, where it occurs in a frequency of 0.03% [8, 9]. Brazilian families with LFS often carry this founder *TP53* germline variant that features a guanine to adenine transition and a subsequent arginine to histidine replacement (c.1010G > A, p.R337H), which confers higher risk of several tumours, specially adrenocortical carcinoma [4]. A recent study proposed that a *XAF1* germline variant, p.E134*, may influence cancer risk and phenotype among p.R337H carriers, given it functions as a tumour suppressor gene that operates in a positive feedback loop with p53 [10]. Pinto *et al* [5] identified that 69% of p.R337H carriers also have the* XAF1* p.E134* variant in the same haplotype, being enriched in patients with cancer, conferring higher risks of sarcoma and multiple primary tumours. Whether the cosegregation of *TP53* and *XAF1* led to the late onset of a G3 pNET in our patient is unknown.

To the authors’ knowledge, there is only one pNET case report associated with LFS, albeit with a different genotype. A 43-year-old Russian and non-Ashkenazi Jewish descendent female with a localised G2 pNET presented a large pancreatic mass (largest diameter was 12.7 cm) which was surgically removed and pathologically diagnosed as a well-differentiated pNET with a ki67 index of 3.5% and a mitotic rate of less than 1 in 50 high-powered fields [6]. Concurrently, the patient was diagnosed with a 6 cm heterogeneous lesion consistent with an angiomyolipoma in her left kidney. During follow-up, she developed a choroid plexus papilloma which was resected. She had a 7-year-old daughter who died from a grade 3 brain astrocytoma with a *TP53* somatic mutation identified in the tumour tissue. The patient was found to carry a pathogenic germline *TP53* variant, c.1009C > T, p.R337C, resulting in arginine to cysteine substitution, which was also present in her pNET tissue with an allele frequency of 93%, strongly suggesting LOH.

Considering Knudson’s two-hit hypothesis, verifying the presence of biallelic inactivation (e.g. inferring LOH by tumour sequencing) can help establish whether PGVs in tumour suppressor genes contributed to cancer development. Both our case and the previously described LFS-associated pNET showed loss of the wild-type *TP53* allele, confirming the biallelic *TP53* inactivation in the tumours. Although only two cases are not enough to confirm a causal association, it is a compelling finding.

Our case report has clinical utility. While the Chompret criteria (e.g. the clinical criteria used to identify suspected LFS individuals who are candidates for genetic testing) does not include the investigation of NET, for individuals with known LFS, routine surveillance with whole body magnetic resonance is probably sufficient to screen for several cancer types, including pNET [6]. Additionally, two prospective cohorts conducted by our group have documented that pNET patients may present PGVs in up to 17% of cases, including *BRCA2*, *CHEK2*, *XPC*, *MUTYH*, *MEN1*, *VHL* and *TSC2* [1–3] and now*, TP53* p.R337. We think patients with early onset pNET, regardless of cancer family history, should be considered for the investigation of PGVs. Such investigations could have tremendous impact on the diagnosis and surveillance of cancer predisposing syndromes, for the pNET patient him/herself, as well as for his/her family members.

Several questions remain: the true penetrance of pNET in *TP53* p.R337H and *XAF1* p.E134* carriers, the role of PGVs in *TP53* residue 337 and pNET onset (both pNET cases developed in carriers of alterations in this amino acid), whether other NETs may arise in this context, the role of *XAF1* mutations as a genetic modifier of *TP53*-R337H/C carriers in the development of pNET, the biological behaviour of LFS-associated pNET and its sensibility to standard NET-directed drugs.

## Conclusion

In conclusion, we report the first case of a pNET in a *TP53* p.R337H and *XAF1* p.E134* carrier. Our case expands on the knowledge of the heterogeneity of LFS, specifically among carriers of the *TP53* p.R337H mutation.

## Conflicts of interest

None to declare.

## Funding

This research was funded by The São Paulo Research Foundation (FAPESP grant numbers 2014/50943-1 and 2018/06269-5), National Council for Scientific and Technological Development (CNPq grant number 465682/2014-6) and Coordination for the Improvement of Higher Education Personnel (CAPES grant number 88887.136405/2017-00).
